# Identification of mRNA Signature for Predicting Prognosis Risk of Rectal Adenocarcinoma

**DOI:** 10.3389/fgene.2022.880945

**Published:** 2022-05-18

**Authors:** Linlin Jiang, Peng Wang, Mu Su, Lili Yang, Qingbo Wang

**Affiliations:** ^1^ Department of Chemotherapy, The Second Hospital of Nanjing, Nanjing University of Chinese Medicine, Nanjing, China; ^2^ Department of General Surgery, The Second Hospital of Nanjing, Nanjing University of Chinese Medicine, Nanjing, China

**Keywords:** rectum adenocarcinoma, mRNA signature, immune, immune infiltrate, prognosis

## Abstract

**Background:** The immune system plays a crucial role in rectal adenocarcinoma (READ). Immune-related genes may help predict READ prognoses.

**Methods:** The Cancer Genome Atlas dataset and GSE56699 were used as the training and validation datasets, respectively, and differentially expressed genes (DEGs) were identified. The optimal DEG combination was determined, and the prognostic risk model was constructed. The correlation between optimal DEGs and immune infiltrating cells was evaluated.

**Results:** Nine DEGs were selected for analysis. Moreover, *ADAMDEC1* showed a positive correlation with six immune infiltrates, most notably with B cells and dendritic cells. *F13A1* was also positively correlated with six immune infiltrates, particularly macrophage and dendritic cells, whereas *LGALS9C* was negatively correlated with all immune infiltrates except B cells. Additionally, the prognostic risk model was strongly correlated with the actual situation. We retained only three prognosis risk factors: age, pathologic stage, and prognostic risk model. The stratified analysis revealed that lower ages and pathologic stages have a better prognosis with READ. Age and mRNA prognostic factors were the most important factors in determining the possibility of 3- and 5-year survival.

**Conclusion:** In summary, we identified a nine-gene prognosis risk model that is applicable to the treatment of READ. Altogether, characteristics such as the gene signature and age have a strong predictive value for prognosis risk.

## Introduction

Rectal adenocarcinoma (READ) is a rare form of colorectal cancer with a high mortality rate worldwide. Patients with early READ who undergo radical surgery have a better prognosis. However, the prognosis for advanced READ is poor, which can endanger the patient’s life and result in death (Burton et al., 2006). Within 2 years, more than 80% of READ patients who experienced local recurrence underwent total mesorectal excision (Chen et al., 2017). Furthermore, after 75 months, no local recurrence of READ necessitating total mesorectal excision was observed (Chen et al., 2017). On average, the 5-year survival rate is described as 66.5% (Fazeli and Keramati 2015). Lymph node and pulmonary metastasis are common in READ, and both contribute to an unfavorable prognosis. Due to the lack of specific characteristics of early READ, early identification has become a considerable challenge. In clinical practice, approximately two-thirds of the READ patients are diagnosed at an advanced stage (Merchea et al., 2018). Typically, noticeable symptoms manifest when the tumor is typically in the middle or late stages. The occurrence of READ is widely believed to be a multistage and multigene process, and tumor occurrence and development of tumors are regulated by several genes (Chen et al., 2017).

Radiation therapy and chemotherapy are frequently used in conjunction with surgery to treat READ patients (Perez et al., 2017). However, these therapies can exacerbate patients’ immune problems. In recent years, immunotherapy as PD-1/PD-L1 immune checkpoints has demonstrated remarkable efficacy in various cancers, including READ (Hecht et al., 2016; Vareki et al., 2017). Tumor immunotherapy aimed to circumvent the tumor immune escape mechanism and awakens the immune cells that are capable of eradicating cancer cells. The immune cells and related genes may play a significant role in the infiltration process. Furthermore, this process has been detected in most human solid tumors, where READ lymphocytic infiltration conferred a survival advantage (Caputo et al., 2016). However, the microenvironment that can predict prognosis in READ remains unknown in terms of molecular events and tumor cell-immunocyte interaction.

Reliable biomarkers can be used to predict prognosis and overall survival. These biomarkers can be clinical variables, physiological or biochemical indicators, or molecular factors (Zhang Z. et al., 2018; Zhang et al., 2019). In recent years, researchers have examined the effect of gene expression levels on predicting survival prognosis for READ patients (Beer et al., 2002). However, most studies were limited by small sample sizes, insufficient evidence, or excessive data. With the development of cancer-specific databases, open and accessible databases such as The Cancer Genome Atlas (TCGA) (Zhu et al., 2018) and ImmPort (Bhattacharya et al., 2018; Sun et al., 2020) provide tremendous and valuable data for mining.

Thus, the gene expression level can be used to deduce specific molecular biological mechanisms underlying tumor occurrence and development. Investigating active and effective tumor markers at the genetic level opens up new treatment options for tumors. We analyzed the transcriptome expression level characteristics of READ samples from The Cancer Genome Atlas database and screened for immune-related genes in READ in this study. In addition, a model for READ disease survival prognostic risk prediction was developed using prognostic-related immune genes.

## Materials and Methods

### Data Source

RNA-seq data from READ patients at the fragments per kilobase million gene level with clinical information and produced by Illumina HiSeq 2000 RNA sequencing platform were downloaded as training datasets from TCGA websites using the genomic data commons data transfer tool (https://gdc-portal.nci.nih.gov/) before 19 October 2021. DEGs were detected using 158 READ tissues and nine normal controls. Supplementary File 1 contains the sample name obtained from TCGA. Simultaneously, the validation data set for GSE56699 was downloaded from the GEO database (http://www.ncbi.nlm.nih.gov/geo/) using the Illumina HumanHT-12 WG-DASL V4.0 R2 expression bead chip. We included the samples with genome-wide expression profile data and clinical prognostic information. Overall, a total of 61 samples were included.

### Identification of Differentially Expressed Genes

The flowchart of this study is depicted in Supplementary Figure S1. The DEGs in the TCGA training data set were identified using the limma package version (v) 3.34.7 of R v3.6.1 (https://bioconductor.org/packages/release/bioc/html/limma.html) with a false discovery rate (FDR) of 0.05 and |log2 (fold change) | > 1. Then, a two-way hierarchical clustering analysis was performed using heatmap v1.0.8 in R v3.6.1 (https://cran.r-project.org/web/packages/pheatmap/index.html) on the DEG expression levels obtained in the training data set.

### Functional Enrichment Analysis of Immune-Related Differentially Expressed Genes

Immune-related DEGs were downloaded for further analysis from the AmiGO 2 (http://amigo.geneontology.org/amigo) and KEGG databases (https://www.kegg.jp/). The function of these DEGs was then determined using the GO biology process and KEGG signal pathway enrichment using DAVID v6.8 (https://david.ncifcrf.gov/), with an FDR threshold of <0.05.

### Construction and Evaluation of the Prognostic Risk Model

Univariate and multivariate Cox regression analyses were used to identify independent DEGs associated with overall survival (OS) using the survival package v2.41-1 of R v3.6.1 (http://bioconductor.org/packages/survivalr/). Significant DEGs were identified using a log-rank *p*-value threshold of <0.05.

The optimal DEGs combination was then determined using the LASSO Cox regression model in R v3.6.1 of penalized package v0.9.50 (https://cran.r-project.org/web/packages/penalized/index.html). The screening model’s optimal parameter “lambda” is obtained through 1,000 cross-validation likelihood algorithm calculation cycles. The following prognostic risk model was constructed using the prognostic coefficients from the LASSO Cox regression model and the DEG expression level:

Prognostic risk score = ∑β_DEGs_ × Exp_DEGs_

Here, β_DEGs_ denote the DEGs coefficient derived from the LASSO Cox regression model, whereas ExpDEGs denote the target DEGs expression level in the training dataset.

### Evaluation of the Prognostic Risk Model

Each sample in the TCGA training and GSE56699 validation datasets was analyzed and assigned a prognostic risk score. The median value was used to classify the samples as high or low risk. In the TCGA training and GSE56699 validation datasets, the correlation between actual survival prognosis and that predicted by the prognostic risk model was evaluated using the Kaplan–Meier curve method in R v3.6.1 with the survival package v2.41-1GSE56699.

### Correlation Analysis Between Prognostic-Related Differentially Expressed Genes and Different Immune Infiltrating Cells

The gene modules of the Tumor Immune Estimation Resource (TIMER; cistrome. shinyapps.io/timer/) were used to investigate the correlation between the expression of prognostic DEGs, and the abundance of six immune infiltrates (B cell, CD4^+^ T cell, CD8^+^ T cell, neutrophil, macrophage, and dendritic cell). A heatmap was generated using the partial correlation index for each DEG in each immune infiltrate and several scatterplots.

### Screening of Independent Clinical Factors

Independent clinical factors such as age (years), gender, pathologic M (M0/M1/-), pathologic N (N0/N1/N2/-), pathologic T (T1/T2/T3/T4/-), pathologic stage (I/II/III/IV/-), history of colon polyps, lymphatic invasion, radiotherapy, prognostic model, death, and OS time (months) screened patients with READ in the TCGA training data set using univariate and multivariate Cox regression analyses with log-rank *p*-value <0.05 as the threshold. The analysis was stratified by age (>65 and ≤65 years of age) and pathologic stage (N0, N1, and N2).

### Model Comparison

In order to evaluate the prognostic risk prediction model, stratified analysis was performed on the samples that were divided into different sample comparing groups. The nomogram displaying 3-year and 5-year OS was constructed to further reveal the correlation between independent factors and actual prognosis using the rms package v5.1-2 (https://cran.r-project.org/web/packages/rms/index.html) in R v3.6.1. survcomp version 1.34.0 (http://www.bioconductor.org/packages/release/bioc/html/survcomp.html) was used to calculate the C-index in R v3.6.1.

## Results

### Identification of Immune-Related Differentially Expressed Genes

**FIGURE 1:**
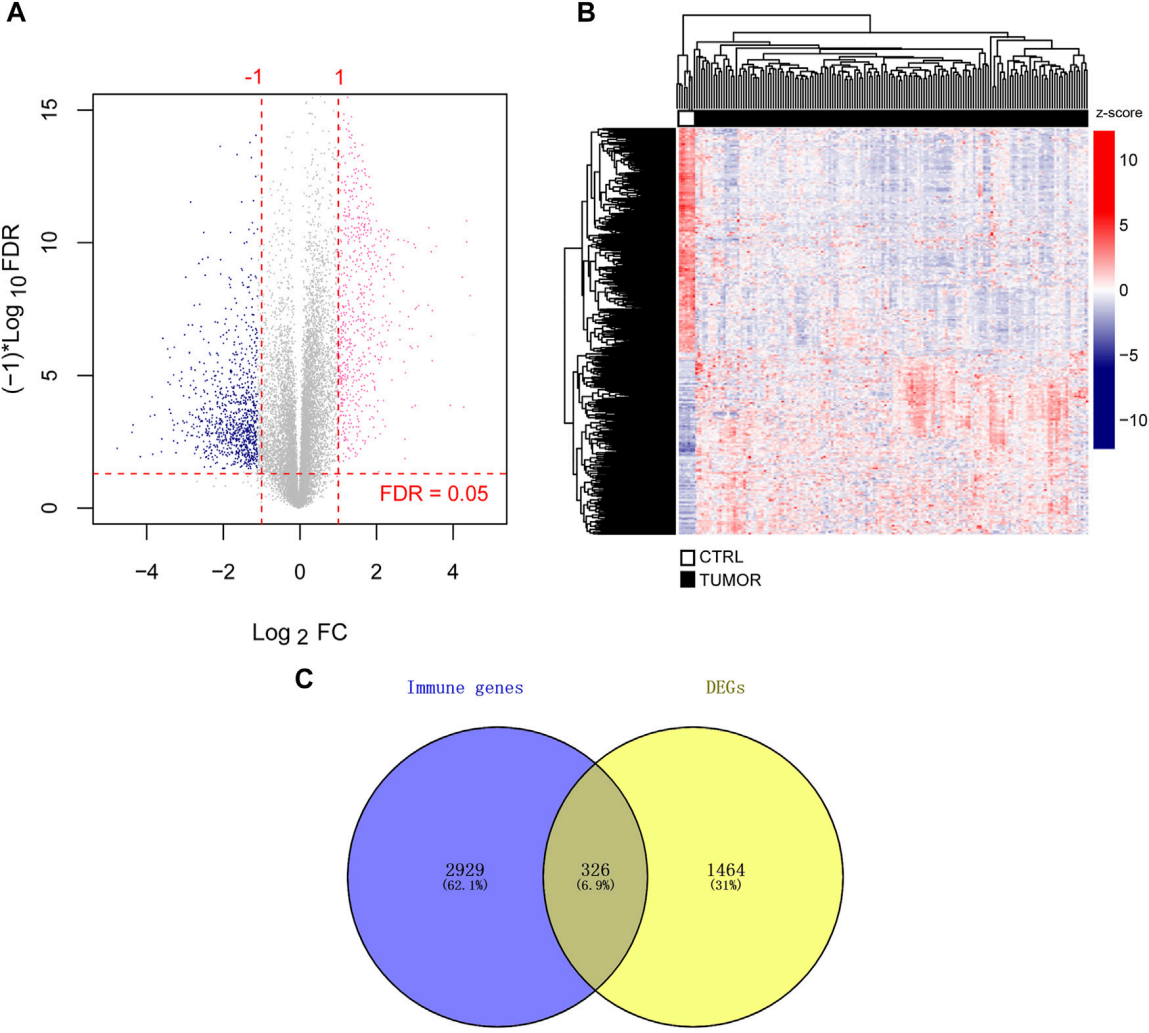
Identification of immune-related DEGs. **(A)** DEGs volcano map. The horizontal axis depicted the effect size (log_2_ FC), while the vertical axis depicted -log10 (FDR). The pink and blue dots represented DEGs that have been upregulated or downregulated, respectively. FDR <0.05 is indicated above the horizontal dashed line, and |log_2_ FC|>1 is indicated outside the two vertical dashed lines. **(B)** Heatmap of DEGs. **(C)** Immune-related genes and DEGs Venn diagram. FDR stands for false discovery rate and DEGs stand for differentially expressed genes. Fold change, FC.

There were 1,772 DEGs identified, with 768 upregulated and 1,004 downregulated across all genes (Figure 1A). From the heatmap, we observed the clustering of tumor and control samples clustered separately, ensuring the reliability of the original data (Figure 1B).

Simultaneously, we downloaded 3,020 and 817 unique immune-related genes from the AmiGO two and KEGG databases, respectively, leaving 3,255 union immune-related genes. When TCGA DEGs were compared to immune-related genes, 326 immune-related DEGs were retained for further investigation (Figure 1C). We provided detailed information on 326 DEGs (log2 FC, *p*-value, and FDR) in Supplementary File 2.

### Functional Enrichment Analysis of Immune-Related Differentially Expressed Genes

**FIGURE 2:**
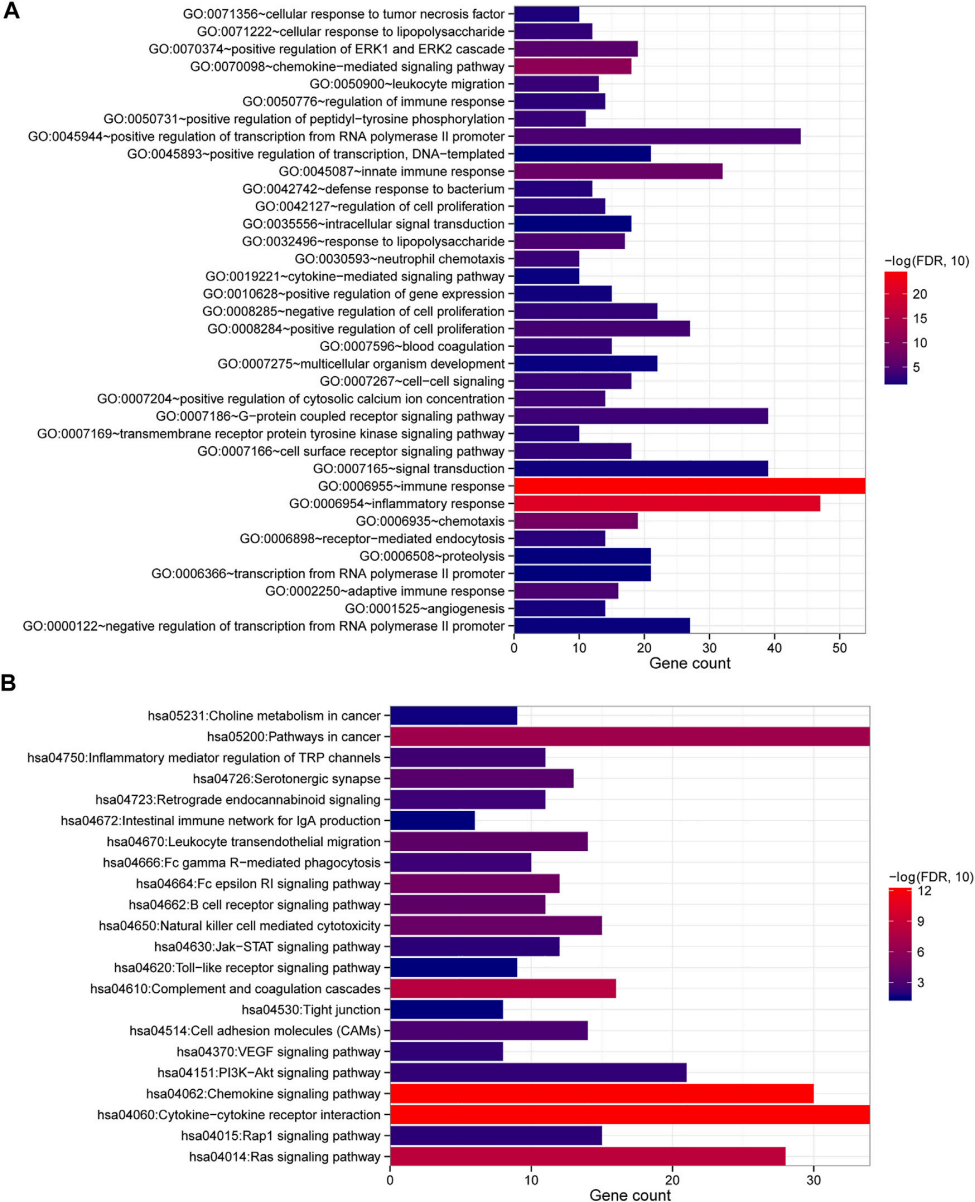
GO and KEGG analysis of DEGs. **(A)** Enriched GO terms with *p* values <0.05. **(B)** KEGG pathways were enriched with a *p* value of <0.05. The number of DEGs was represented by the horizontal axis, and the GO or KEGG items were represented by the vertical axis: the greater the significance, the closer the column color is to red.

Additionally, we examined the functions of 326 DEGs using GO and KEGG analyses, identifying 36 BP and 22 KEGG under a <0.05 *p*-value (Figure 2; Table 1). The DEGs were mainly enriched in the GO term of the immune response, inflammatory response, chemokine-mediated signaling pathway, chemotaxis, innate immune response, positive regulation of the ERK1 and ERK2 cascades, response to lipopolysaccharide, adaptive immune response, positive regulation of transcription from the RNA polymerase II promoter, and positive regulation of cell proliferation (Figure 2A). Additionally, KEGG pathways also involved cytokine–cytokine receptor interactions; the chemokine signaling, Ras signaling, complement, coagulation cascades, cancer, Fc epsilon RI signaling pathways; natural killer cell-mediated cytotoxicity; leukocyte transendothelial migration; B cell receptor signaling pathway, and serotonergic synapse (Figure 2B).

**TABLE 1:** Top ten GO and KEGG analyses on 326 DEGs.

ID	Term	Count	*p* value	FDR
Significantly enriched GO terms on biology process (*p* < 0.05)				
0006955	Immune response	54	5.14E-29	1.19E-25
0006954	Inflammatory response	47	1.54E-24	1.79E-21
0070098	Chemokine-mediated signaling pathway	18	1.02E-14	7.87E-12
0006935	Chemotaxis	19	1.12E-11	6.49E-09
0045087	Innate immune response	32	1.16E-10	5.38E-08
0070374	Positive regulation of ERK1 and ERK2 cascade	19	4.60E-09	1.78E-06
0032496	Response to lipopolysaccharide	17	7.10E-08	2.35E-05
0002250	Adaptive immune response	16	1.10E-07	3.20E-05
0045944	Positive regulation of transcription from RNA polymerase II promoter	44	1.37E-07	3.53E-05
0008284	Positive regulation of cell proliferation	27	6.57E-07	1.17E-04
Significantly enriched KEGG pathways (p < 0.05)				
hsa04060	Cytokine–cytokine receptor interaction	34	7.74E-15	6.88E-13
hsa04062	Chemokine signaling pathway	30	1.02E-14	6.88E-13
hsa04014	Ras signaling pathway	28	6.01E-11	2.70E-09
hsa04610	Complement and coagulation cascades	16	3.16E-10	8.52E-09
hsa05200	Pathways in cancer	34	4.69E-09	1.05E-07
hsa04664	Fc epsilon RI signaling pathway	12	1.87E-06	3.60E-05
hsa04650	Natural killer cell-mediated cytotoxicity	15	5.15E-06	8.68E-05
hsa04670	Leukocyte transendothelial migration	14	1.33E-05	1.80E-04
hsa04662	B-cell receptor signaling pathway	11	1.53E-05	1.88E-04
hsa04726	Serotonergic synapse	13	4.53E-05	4.70E-04

FDR, false discovery rate; GO, Gene Ontology; KEGG, Kyoto Encyclopedia of Genes and Genomes.

### Construction and Evaluation of the Prognostic Risk Model

The overall 326 DEGs were subjected to univariate Cox regression analysis, and 41 were identified as prognostic-related DEGs. Following the multivariate Cox regression analysis, a total of 22 DEGs remained. After that, nine optimal DEG combinations related to immunity were identified, including galectin 9C [LGALS9C; hazard ratio (HR) = 0.930, 95% confidence interval (CI) = 0.850–0.973], coagulation factor XIII A chain (*F13A1*; HR = 1.012, 95% CI = 1.004–1.554), ADAM-like decysin 1 (*ADAMDEC1*; HR = 0.987, 95% CI = 0.854–0.992), macrophage receptor with collagenous structure (*MARCO*; HR = 1.008, 95% CI = 1.002–1.504), L3MBTL histone methyl-lysine binding protein 1 (*L3MBTL1*; HR = 0.883, 95% CI = 0.803–0.970), solute carrier family 7 member 11 (*SLC7A11*; HR = 0.881, 95% CI = 0.814–0.954), UL16 binding protein 3 (*ULBP3*; HR = 0.884, 95% CI = 0.800–0.976), complement component 4 binding protein alpha (*C4BPA*; HR = 1.019, 95% CI = 1.001–1.458), and Cbp/p300 interacting transactivator with Glu/Asp rich carboxy-terminal domain 1 (*CITED1*; HR = 1.065, 95% CI = 1.008–1.125) (Table 2). The prognostic risk model was constructed based on *LGALS9C*, *F13A1*, *ADAMDEC1*, *MARCO*, *L3MBTL1*, *SLC7A11*, *ULBP3*, *C4BPA*, and *CITED1* expression levels. Then, using the constructed model, the prognostic risk score of each sample was calculated as: prognostic risk score = (−0.2332) × Exp*LGALS9C* + 0.1177 × Exp*F13A1* + (−0.1821) × Exp_*ADAMDEC1*_ + 0.1287 × Exp_MARCO_+ (−0.4846) × Exp_L3MBTL1_+ (−0.6165) × Exp_SLC7A11_+ (−0.5122) × Exp_ULBP3_+ 0.0929 × Exp_C4BPA_+ 0.3338 × Exp_CITED1_.

**TABLE 2:** Optimal combination immune-related DEGs.

Symbol	HR	95%CI	*p*	LASSO coef
*LGALS9C*	0.930	0.850–0.973	1.18E-02	−0.2332
*F13A1*	1.012	1.004–1.554	4.58E-02	0.1177
*ADAMDEC1*	0.987	0.854–0.992	4.67E-02	−0.1821
*MARCO*	1.008	1.002–1.504	4.71E-02	0.1287
*L3MBTL1*	0.883	0.803–0.970	9.52E-03	−0.4846
*SLC7A11*	0.881	0.814–0.954	1.78E-03	−0.6165
*ULBP3*	0.884	0.800–0.976	1.45E-02	−0.5122
*C4BPA*	1.019	1.001–1.458	1.76E-02	0.0929
*CITED1*	1.065	1.008–1.125	2.44E-02	0.3338

CI, confidence interval; HR, hazard ratio.

### Correlation Analysis Between the Expression of Prognostic-Related DEGs and Six Immune Infiltrates

**FIGURE 3:**
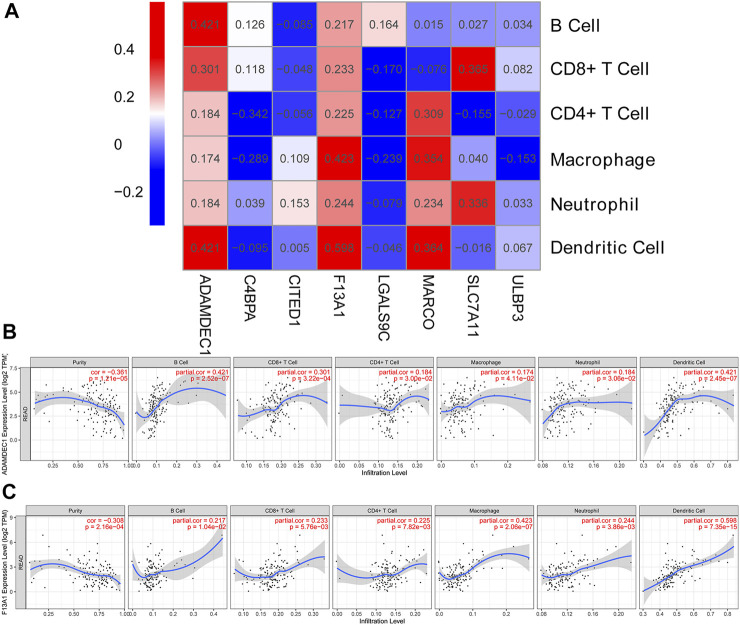
Correlation heatmap between DEGs and immune infiltration cells. **(A)** Heatmap of the correlation between DEGs and immune infiltration cells. **(B,C)** Scatter plots of the correlation between immune infiltration cells and *ADAMDEC1* and *F13A1* expression levels, respectively.

The highly expressed genes in tumor cells typically have positive associations with tumor purity. As such, we examined the association between prognosis DEG expression and six immune infiltrates (Supplementary Figure S2). *ADAMDEC1* correlated positively with six immune infiltrates, particularly B cell and dendritic cell infiltrates (partial correlation = 0.421, *p* = 2.52e-07; Figures 3A,B). The results indicated that increased *ADAMDEC1* expression was associated with a higher purity of READ tumor cells in B and dendritic cells. *F13A1* also had positive correlation with six immune infiltrates, most notably macrophages (partial correlation = 0.423, *p* = 2.06e-07; Figures 3A,C) and dendritic cells (partial correlation = 0.598, *p* = 7.35e-15; Figures 3A,C). Apart from the B cells, *LGALS9C* had a negative correlation with immune infiltrates (Figure 3A).

### Evaluation of the Prognostic Risk Model

**FIGURE 4:**
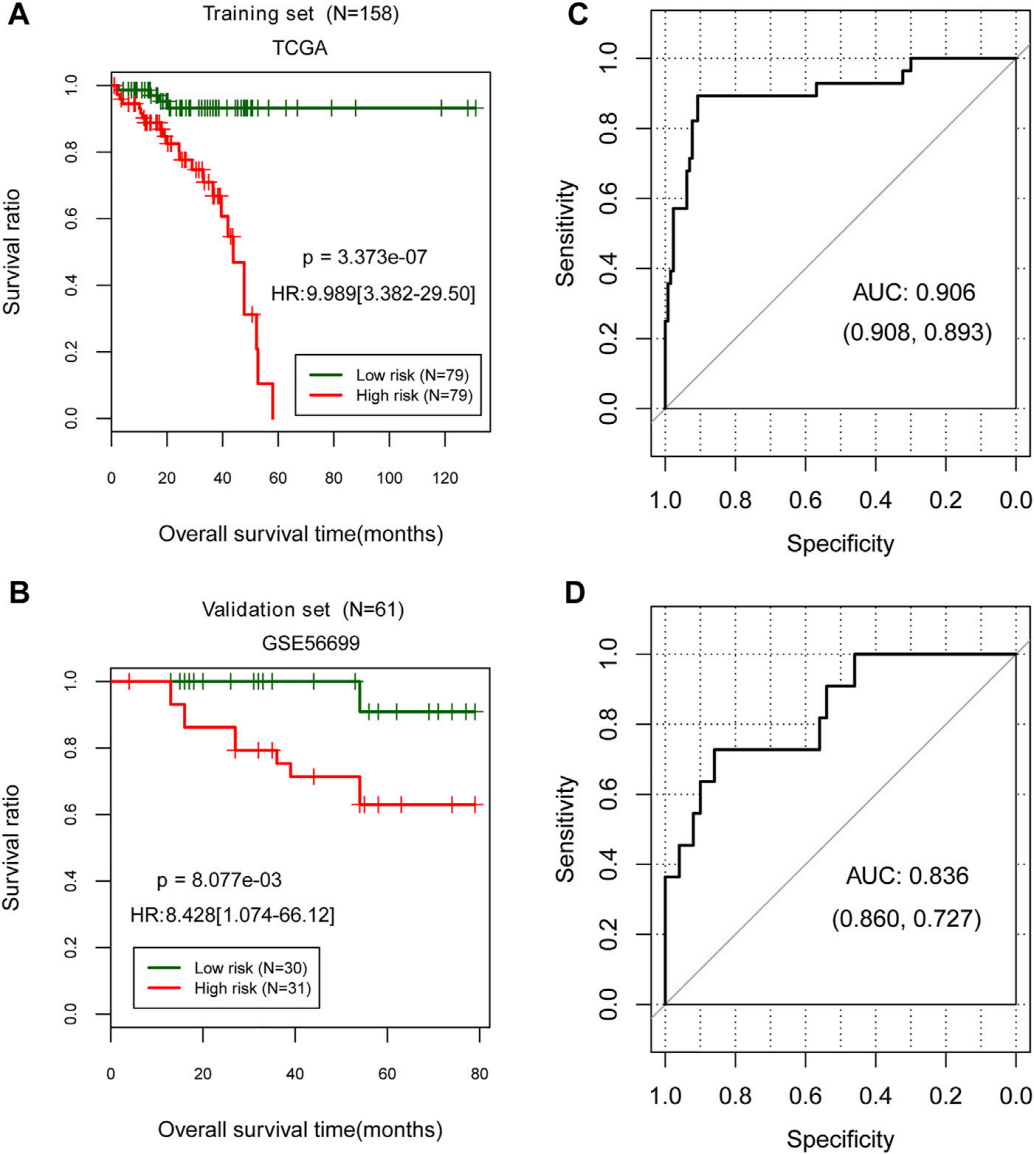
Evaluation of the prognostic risk model in TCGA training data set and GSE56699 validation dataset. **(A,C)** Kaplan–Meier curve method was used to evaluate a prognostic risk model in the TCGA training and GSE56699 validation datasets. The ROC curve of the prognostic risk model prediction results **(B,D)**. Numbers in parentheses in the figure represent the ROC curve’s specificity and sensitivity.

The prognostic risk model was evaluated by classifying samples into high- and low-risk groups in the TCGA training and GSE56699 validation datasets. Then, in two data sets, the high- and low-risk groups were compared according to their prognostic risk models to those classified according to their actual status. Both TCGA training [HR = 9.989 (3.382–29.50), *p* = 3.373e-07; Figure 4A] and GSE56699 validation datasets [HR = 8.428 (1.074–66.12), *p* = 8.077e-03; Figure 4B] revealed significant differences between high- and low-risk groups. Additionally, the prognostic risk model demonstrated a strong correlation with the actual situation in both the TCGA training [AUC = 0.906 (0.908, 0.893); Figure 4C] and GSE56699 validation datasets [AUC = 0.836 (0.860, 0.727); Figure 4D].

### Screening of Independent Clinical Factors

Univariable Cox regression analysis was used to eliminate independent clinical prognosis factors such as age (*p* = 7.97e-05), pathologic M (*p* = 2.30e-03), pathologic N (*p* = 4.61e-03), pathologic T (*p* = 3.07e-02), pathologic stage (*p* = 1.03e-03), and prognostic model (*p* = 3.37e-07) (Table 3). After multivariable Cox regression analysis, only three factors were retained for further investigation: age (*p* = 1.27e-03), pathologic stage (*p* = 4.91e-02), and prognostic risk model (*p* = 4.88e-03; Table 3).

**TABLE 3:** Independent clinical factor selection.

Clinical characteristic	TCGA (N = 158)	Univariable Cox			Multivariable Cox		
		HR	95% CI	*p*	HR	95% CI	*p*
Age (years, mean ± sd)	64.21 ± 11.42	1.101	1.048–1.157	**7.97E-05**	1.112	1.042–1.186	**1.27E-03**
Gender (male/female)	89/69	0.878	0.416–1.851	7.31E-01	-	-	-
Pathologic_M (M0/M1/-)	119/23/16	3.381	1.472–7.767	**2.30E-03**	3.304	0.628–17.39	1.58E-01
Pathologic_N (N0/N1/N2/-)	80/43/31/4	1.886	1.195–2.976	**4.61E-03**	2.559	1.004–6.527	**4.91E-02**
Pathologic_T (T1/T2/T3/T4/-)	9/28/106/13/2	2.127	1.034–4.379	**3.07E-02**	1.464	0.605–3.539	3.98E-01
Pathologic_stage (I/II/III/IV/-)	30/47/47/24/10	2.131	1.328–3.419	**1.03E-03**	1.460	0.642–2.491	1.95E-01
History of colon polyps (yes/no/-)	30/109/19	1.003	0.336–2.992	9.95E-01	-	-	-
Lymphatic invasion (yes/no/-)	57/83/18	1.158	0.499–2.686	7.33E-01	-	-	-
Radiotherapy (yes/no/-)	20/107/31	0.422	0.249–7.638	9.98E-01	-	-	-
Prognostic risk model (high/low)	79/79	9.989	3.382–29.50	**3.37E-07**	9.067	1.953–42.03	**4.88E-03**
Death (yes/no)	28/130	-	-	-	-	-	-
Overall survival time (months, mean ± sd)	27.05 ± 21.57	-	-	-	-	-	-

Note: the P-value <0.05 was bold.

**FIGURE 5:**
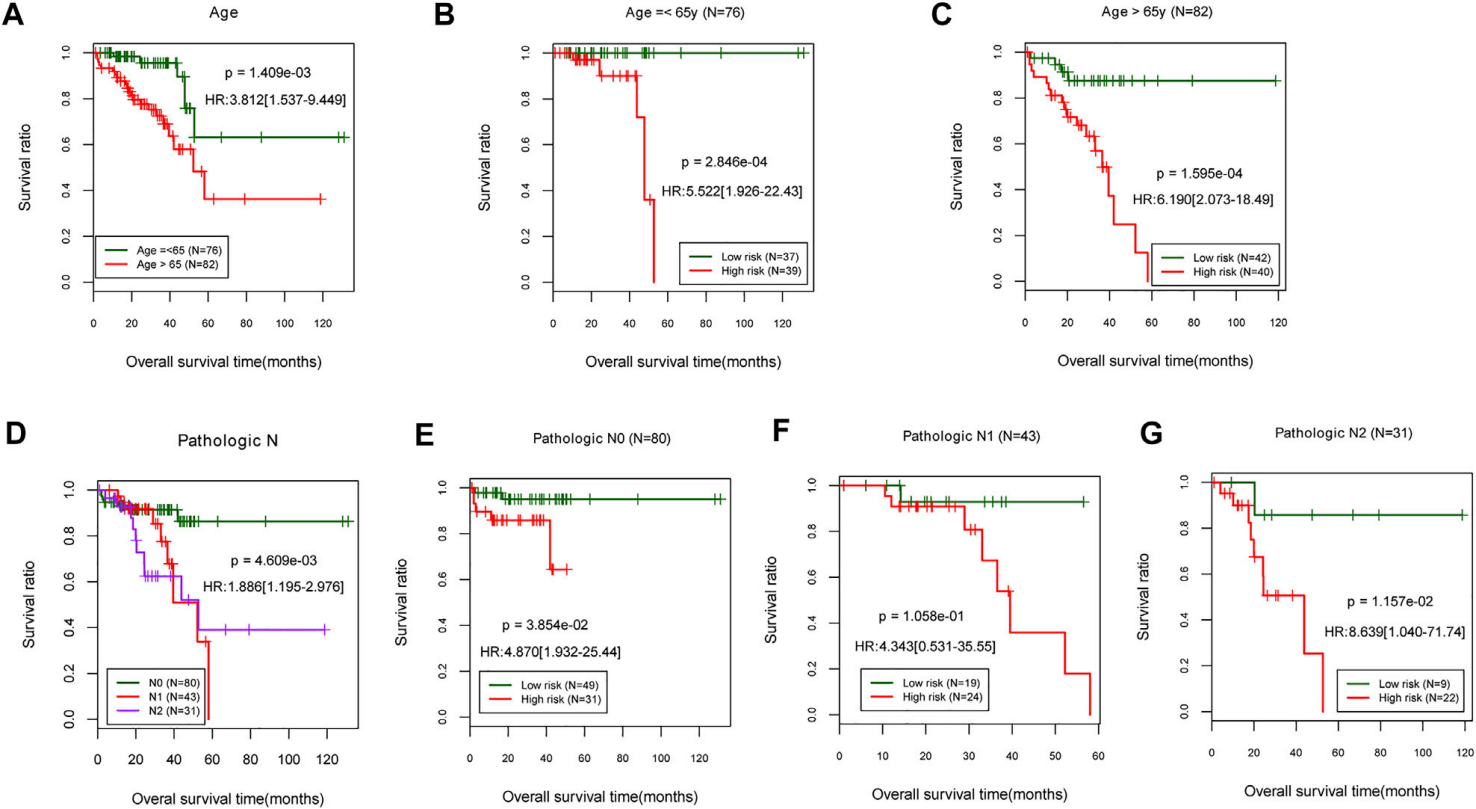
Stratified analysis on age and pathologic. **(A)** Age-related prognostic Kaplan–Meier curve. **(B,C)** Prognosis-related Kaplan–Meier curves in TCGA samples for patients aged 65 and younger. **(D)** Prognostic-related Kaplan–Meier curve of pathologic stage. The pathologic stages N0, N1, and N2 are represented in TCGA sample’s prognosis-related Kaplan–Meier curve chart **(E–G)**. TCGA, The Cancer Genome Atlas.

The stratified analysis based on age (>65 and ≤65 years old) revealed that patients aged 65 years and older had a significantly lower survival rate [HR = 3.812 (1.537–9.449), *p* = 1.409e-03; Figure 5A]. According to the risk score proposed above, the samples in each subgroup were divided into low- and high-risk groups. In patients ≤65 years of age, those with high-risk scores had a significantly shorter OS time than those with low-risk scores [HR = 5.522 (1.926–22.43), *p* = 2.846e-04; Figure 5B]. In patients over the age of 65, those with a high-risk score had a significantly shorter OS time than those with a low-risk score [HR = 6.190 (2.073–18.49), *p* = 1.595e-04; Figure 5C].

The stratified analysis based on pathologic stage (N0, N1, and N2) revealed that patients with a higher pathologic stage [N1, N2; HR = 1.886 (1.195–2.976), *p* = 4.609e-03; Figure 5D] had a significantly lower survival rate than those who were (N0). The patients with low-risk scores at pathologic N0 have a significantly shorter OS time than patients with high-risk scores [HR = 4.870 (1.932–25.44), *p* = 3.854e-02; Figure 5E]. There were no significant differences in OS times between pathologic N1 and N2 patients classified as high- or low-risk (Figures 5F,G). The results indicated that a lower age (≤65 years) and a more advanced pathologic stage are associated with a better prognosis for READ patients, consistent with their actual status.

### Model Comparison

**FIGURE 6:**
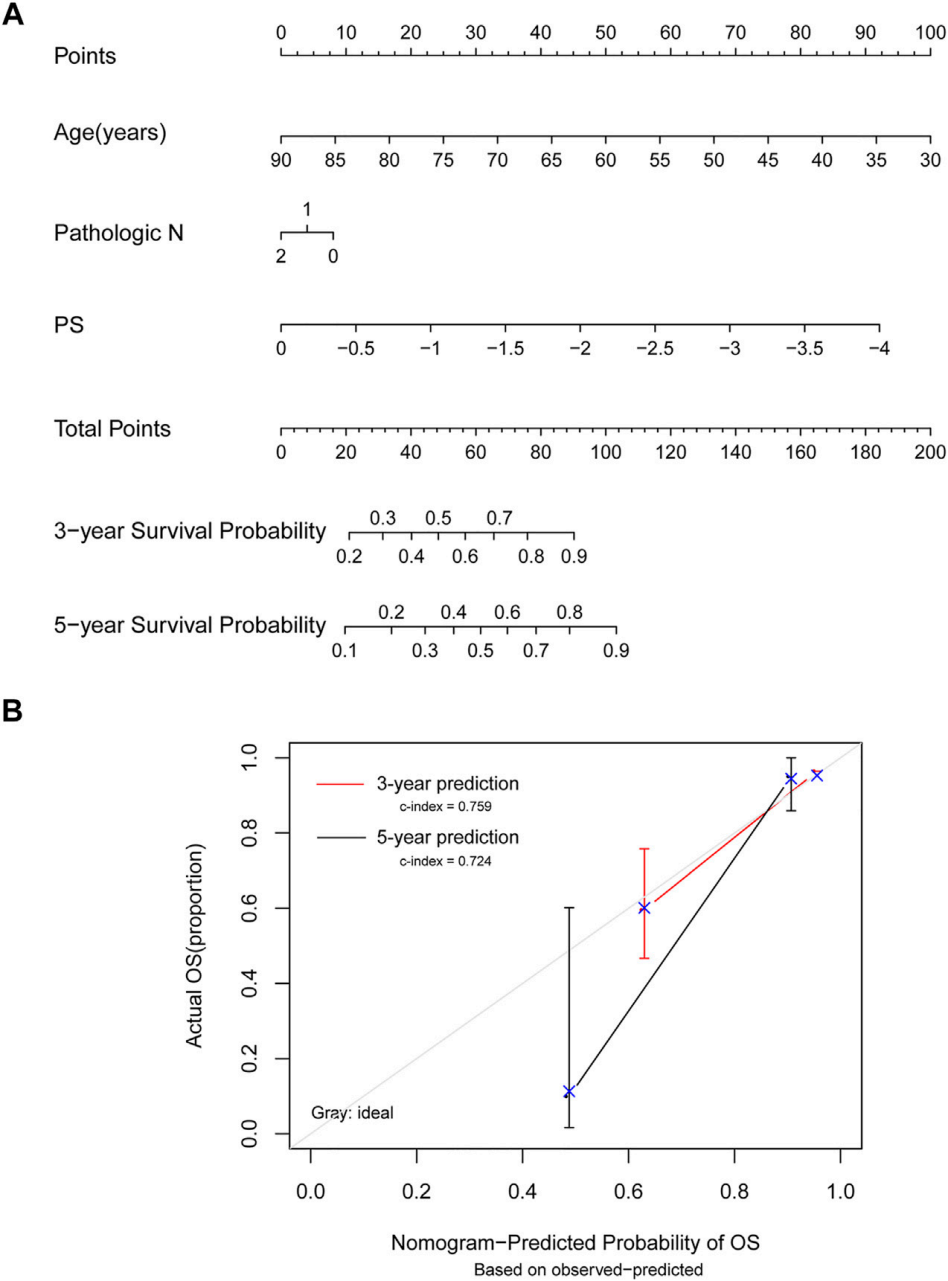
Model comparison analyses. **(A)** Nomogram survival rate prediction model for independent prognostic factors. **(B)** A 3-year and 5-year survival rate prediction line graph and an actual survival rate consistency line graph. The horizontal axis shows the predicted OS rate, the vertical axis shows the actual OS rate, and the red and black lines show 3- and 5-year predicted line graphs, respectively.

The survival nomogram model analysis of TCGA training dataset samples revealed that age and mRNA prognostic factors were the most significant predictors of 3- and 5-year survival (Figure 6A). The 3-year (C-index = 0.759) and 5-year (C-index = 0.724) survival probabilities predicted by the model were generally consistent with actual survival rates (Figure 6B).

## Discussion

Age, gender, and TNM stage are frequently used as prognostic factors in most cancers, including READ. Nevertheless, the high heterogeneity and limited predictive capacity of READ necessitate the inclusion of additional prognosis biomarkers. TCGA has recently provided robust data support for data reanalysis. The use of mRNA signatures can help accelerate the development and application of tumor-specific diagnostic technology, aid in the development of anti-tumor biologics at the genetic level and provide new avenues for tumor treatment. The immune system plays a critical role in the development of all cancers. The local interactions between tumor cells and immune cells, and endothelial and stromal cells, have been shown to have both pro- and anti-tumor effects (Braun et al., 2019). Therefore, we focused on immune-related genes in READ. This study sought to determine the effect of immune-related genes on the prognosis of READ and observed their expression in immune cells.

The present study identified a nine-gene immune-related mRNA signature biomarker. These genes LGALS9C, *F13A1*, *ADAMDEC1*, MARCO, L3MBTL1, SLC7A11, ULBP3, C4BPA, and *CITED1* were included in the READ prognosis risk model. As an isoform of LGALS9, *LGALS9C* is a class of several eosinophil chemoattractants produced by activated T cells (Sato et al., 2002). Notably, these chemoattractants had previously been identified at multiple immune checkpoints (Huang X. et al., 2020). Also, it was discovered that this checkpoint was involved in the prognosis and therapeutic efficacy of READ in the current study. Additionally, unlike the B cells, *LGALS9C* exhibited a negative correlation with immune infiltrates. High LGALS9 scores were found in every immune subtype, although they were higher in the immune-rich tumors (Alame et al., 2021).

According to Luo et al. (2020), *F13A1* alters the immune response and increases the risk of postoperative recurrence in cancers. When combined with BAMBI and LCN2, *F13A1* demonstrated superior prognostic properties than when it is used alone (Luo et al., 2020). Additionally, it has been implicated in the development and progression of cancer (Vairaktaris et al., 2007). *F13A1* has also been linked to lung cancer (Gao et al., 2019). In this study*, F13A1 was identified as* an important immune-related gene with a positive correlation to six immune infiltrates, particularly macrophage and dendritic cells. As previously reported, *F13A1* inhibits preadipocyte proliferation by downregulating the downstream proliferative signaling pathways and defaulting to hypertrophic adipocyte differentiation profiles as an antagonistic.

*ADAMDEC1* is a unique metazinc metalloprotease belonging to the A disintegrin and metalloproteases (ADAMs) family. Furthermore, the studies revealed that *ADAMDEC1*, which is required for the interaction of dendritic cells and germinal center T-helper cells (Fritsche et al., 2003), was involved in protein metabolism and cell adhesion during preoperative radiotherapy for rectal cancer (Supiot et al., 2013). Additionally, it has been associated with a variety of inflammatory diseases, including atherosclerosis (Papaspyridonos et al., 2006), pulmonary sarcoidosis (Papaspyridonos et al., 2006), osteoarthritis (Papaspyridonos et al., 2006), Crohn’s disease (de Bruyn et al., 2014), gastric adenocarcinoma (Pasini et al., 2014), and colorectal cancer (Macartney-Coxson et al., 2008). Additionally, we discovered a positive correlation between *ADAMDEC1* and six immune infiltrates, most notably B and dendritic cells. On binding to PU.1, *ADAMDEC1* expression can be regulated in activated dendritic cells and macrophages. The macrophages and B cells express PU.1, which is required for myeloid cell differentiation (Klemsz et al., 1990; Valledor et al., 1998). As reported by (Tong et al., 2021), high *ADAMDEC1* expression was significantly correlated with better prognosis. *MARCO* was also identified as one of six diagnostic and prognostic biomarkers for patients with lung adenocarcinoma (Shang et al., 2017). However, to our knowledge, no previous studies have established *MARCO* as a READ biomarker. In addition, L3MBTL1 has been identified as a prognosis gene associated with a low risk of recurrence in low-grade, hormone receptor-positive tumors (Wismar et al., 1995). Moreover, we combined L3MBTL1 with hsa-miRNA-595 and lncRNA RP11-909B2.1 to develop a viable biomarker panel for colorectal cancer diagnosis and prognosis.

As shown in our present study, L3MBTL1 functions as both a biomarker for colorectal cancer and READ. Similarly, our previous study demonstrated that overexpressed *SLC7A11* was validated as an oncogene in hepatocellular carcinoma (Zhang L. et al., 2018). Additionally, it has been suggested that it may be a prognostic gene in hepatocellular carcinoma (Yue et al., 2019). The previous reports also stated that CITED1 is correlated with lymph node metastasis in CRC patients, suggesting that it may be used to predict the presence of lymph node metastasis (Nasu et al., 2013). Furthermore, *CITED1* knockdown can lead to decreased cellular proliferation and modulation of several genes (Rogers et al., 2016). Few studies have examined the effects of *ULBP3* or *C4BPA* on the prognosis of READ. Most of these nine genes were associated with prognosis or cancer, and none were identified as READ biomarkers. Our present study revealed a new perspective on READ, which may play an important role in READ prognoses.

Likewise, Zuo et al. (2019) identified a six-gene signature (*EPHA6*, *TIMP1*, *IRX6*, *ART5*, *HIST3H2BB*, and *FOXD1*) for predicting the prognosis of READ, while (Huang W. et al. (2020) identified a novel mRNA panel for READ prognosis prediction and risk stratification. However, these studies did not examine the relationship between the pathogenesis or progression of READ and the immune system, which may be critical in treatment. Thus, identifying novel and meaningful biomarkers associated with immune-related genes is crucial for the prognosis and treatment of READ patients (Zhang et al., 2015a; Zhang, et al., 2015b). Our present study focused on the immune-related genes and identified significant biomarkers for prognosis prediction.

In summary, we identified an immune-related prognosis risk model that may be useful in the treatment of READ.

## Data Availability

The original contributions presented in the study are included in the article/Supplementary Material, further inquiries can be directed to the corresponding author.

## Supplementary Material

Supplementary Figure S1 Flowchart of this study.

Supplementary Figure S2 Association between prognosis DEG expression and six immune infiltrates.
