# Supplementary figures and images for: Identification of mRNA Signature for Predicting Prognosis Risk of Rectal Adenocarcinoma
Source: Front Genet. 2022 May 18;13:880945. doi: 10.3389/fgene.2022.880945 (PMC9159392; doi:10.3389/fgene.2022.880945)

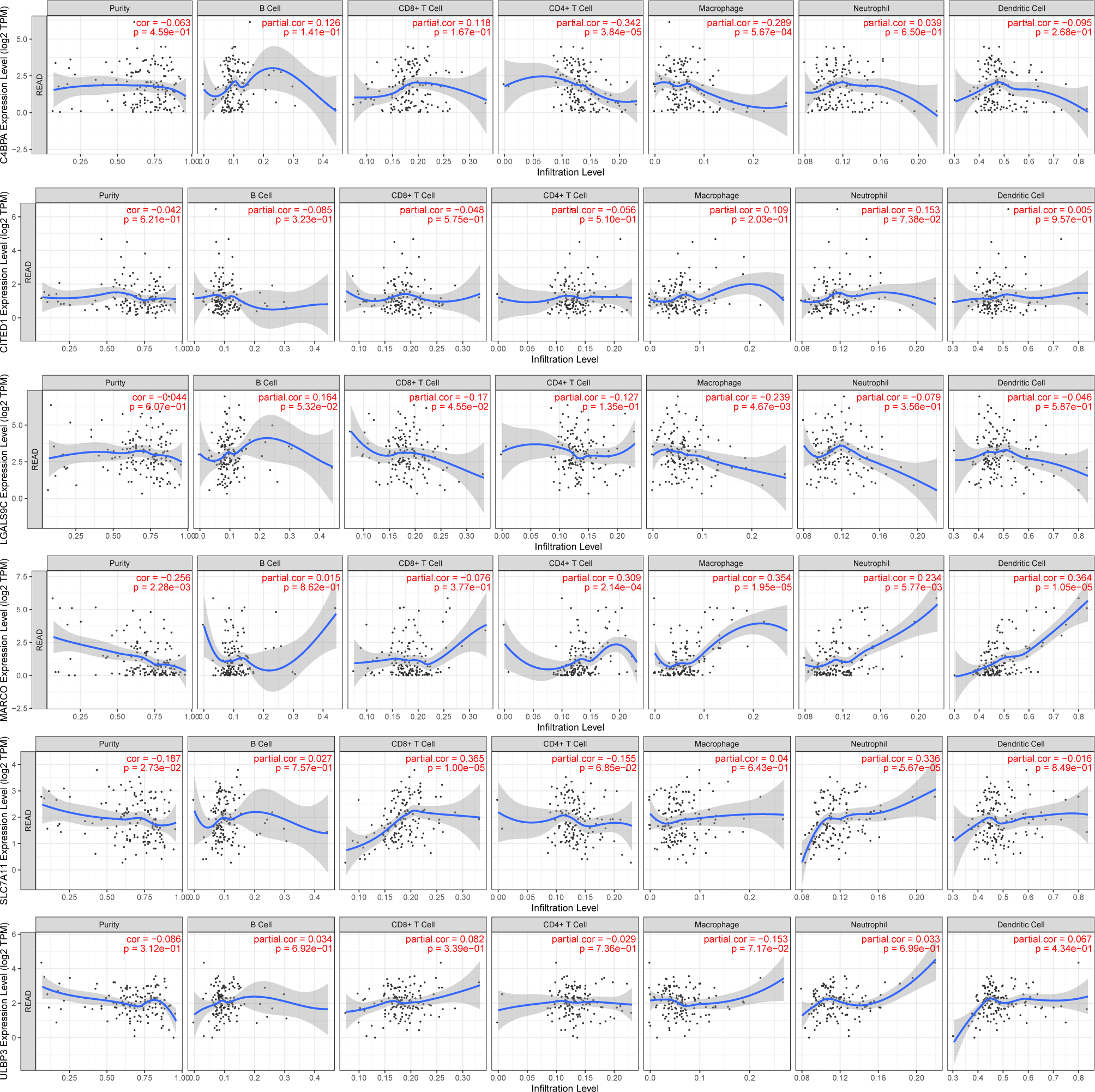

Supplement: Supplementary file 2 [file Image2.tif]

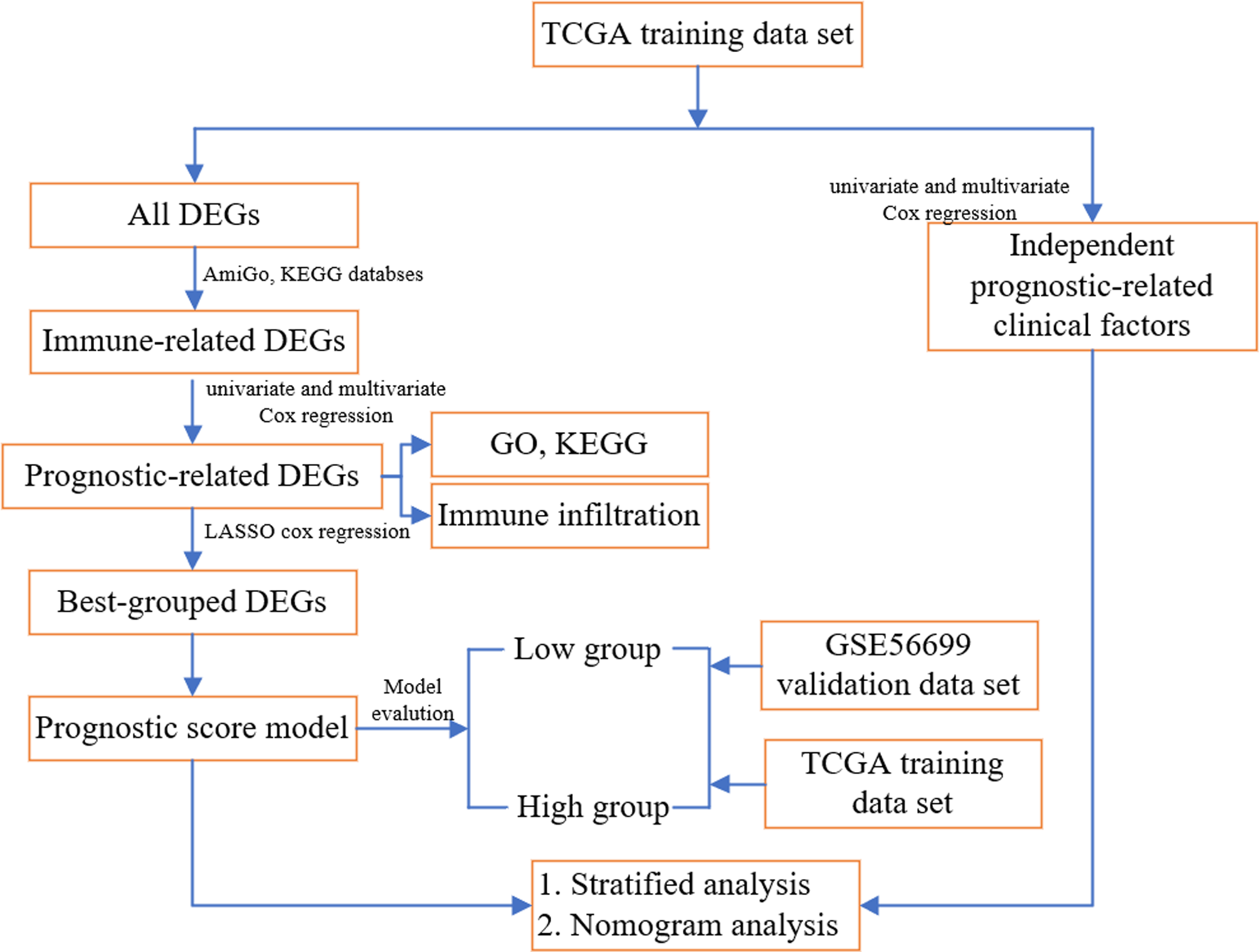

Supplement: Supplementary file 3 [file Image1.tif]
